# Inhibition of Sphingosine-1-Phosphate Receptor 2 by JTE013 Enhanced Alveolar Bone Regeneration by Promoting Angiogenesis

**DOI:** 10.3390/ijms24043401

**Published:** 2023-02-08

**Authors:** William Lory, Bridgette Wellslager, Chao Sun, Özlem Yilmaz, Hong Yu

**Affiliations:** 1 Department of Oral Health Sciences, College of Dental Medicine, Medical University of South Carolina, Charleston, SC 29425, USA; 2 Division of Laboratory Animal Resources, Medical University of South Carolina, Charleston, SC 29425, USA

**Keywords:** *S1PR2*, JTE013, *VEGF*, *PDGF*, *GDF15*, angiogenesis, bone regeneration

## Abstract

Sphingosine-1-phosphate receptor 2 (*S1PR2*) is a G protein-coupled receptor that regulates various immune responses. Herein, we report the effects of a *S1PR2* antagonist (JTE013) on bone regeneration. Murine bone marrow stromal cells (BMSCs) were treated with dimethylsulfoxide (DMSO) or JTE013 with or without infection by an oral bacterial pathogen *Aggregatibacter actinomycetemcomitans*. Treatment with JTE013 enhanced vascular endothelial growth factor A (*VEGFA*), platelet derived growth factor subunit A (*PDGFA*), and growth differentiation factor 15 (*GDF15*) gene expression and increased transforming growth factor beta (TGFβ)/Smad and Akt signaling. Eight-week-old male C57BL/6J mice were challenged with ligatures around the left maxillary 2nd molar for 15 days to induce inflammatory bone loss. After ligature removal, mice were treated with diluted DMSO or JTE013 in the periodontal tissues 3 times per week for 3 weeks. Calcein was also injected twice to measure bone regeneration. Micro-CT scanning of maxillary bone tissues and calcein imaging revealed that treatment with JTE013 enhanced alveolar bone regeneration. JTE013 also increased *VEGFA*, *PDGFA*, osteocalcin, and osterix gene expressions in the periodontal tissues compared to control. Histological examination of periodontal tissues revealed that JTE013 promoted angiogenesis in the periodontal tissues compared to control. Our findings support that inhibition of *S1PR2* by JTE013 increased TGFβ/Smad and Akt signaling; enhanced *VEGFA*, *PDGFA*, and *GDF15* gene expression; and subsequently promoted angiogenesis and alveolar bone regeneration.

## 1. Introduction

**Figure 1:**
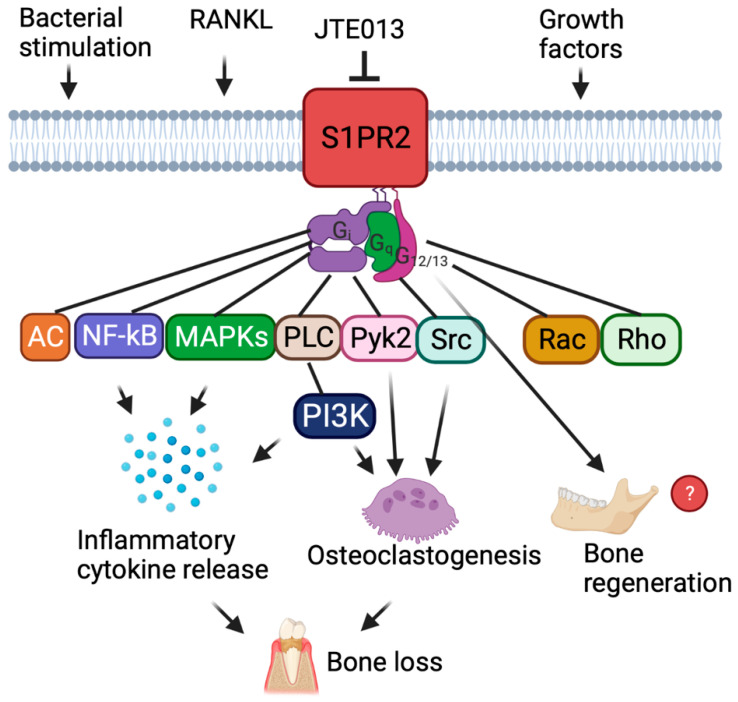
Schematic illustration of the role of sphingosine-1-phosphate receptor 2 (S*1PR2*). S*1PR2* is a transmembrane receptor that couples with G_i_, G_q_, and G_12/13_ proteins and regulates various cellular signaling pathways, including adenylate cyclase (AC), phospholipase C (PLC), phosphoinositide-3 kinase (PI3K), nuclear kappa-B (NF-κB), mitogen-activated kinases (MAPKs), protein tyrosine kinase 2 (Pyk2), proto-oncogene tyrosine protein kinase Src (Src), and the small G proteins Rac and Rho. JTE013 is a specific *S1PR2* antagonist. Bacteria stimulate *S1PR2*, which activates NF-κB, PI3K, and MAPKs signaling pathways, leading to the release of inflammatory cytokines. RANKL stimulates *S1PR2*, which activates PI3K, Pyk2, and Src sigaling pathways, resulting in osteoclastogenesis and bone resorption. It is unknown if *S1PR2* controls bone regeneration.

Sphingosine-1-phosphate receptor 2 **(***S1PR2*) is one of the five G protein-coupled S1P receptors (S1PR1–5). *S1PR2* couples with heterotrimeric G_i_, G_q_, and G_12/13_ proteins and regulates various cellular signaling pathways, including adenylate cyclase (AC), phospholipase C (PLC), phosphoinositide-3 kinase (PI3K), nuclear kappa-B (NF-κB), mitogen-activated kinases (MAPKs), and the small G proteins Rac and Rho (Figure 1) [1,2,3,4]. Previous studies demonstrated that *S1PR2* not only controls S1P signaling, but also regulates inflammatory responses induced by various stimuli [5,6,7,8,9]. Moreover, inhibition of *S1PR2* by a *S1PR2*-specific antagonist (JTE013) reduced serum IL-1β and IL-18 levels in mice when challenged by bacterial lipopolysaccharide (LPS) [5]. Additionally, treatment with JTE013 in mice alleviated colitis induced by deoxycholic acid and dextran sulfate sodium [6] and attenuated lung inflammation stimulated by ovalbumin [7]. In our previous studies using murine bone marrow-derived monocytes and macrophages (BMMs), knockdown of *S1PR2* by a *S1PR2* shRNA or pharmacological inhibition of *S1PR2* by JTE013 decreased the levels of IL-1β, IL-6, and TNF-α inflammatory cytokines induced by the oral bacterial pathogen *Aggregatibacter actinomycetemcomitans* (*Aa*) via attenuating bacteria-induced PI3K, NF-κB, and MAPKs signaling [8,9]. Additionally, we demonstrated that treatment with the *S1PR2* shRNA or JTE013 suppressed osteoclastogenesis induced by RANKL by down-regulating podosome components (basic cell adhesive units, including PI3K, Pyk2, Src, F-actin, integrin β3, and paxillin levels) [8,9]. In a ligature-induced periodontitis animal model, we also demonstrated that treatment with JTE013 alleviated periodontal inflammation and osteoclastogenesis, subsequently reducing alveolar bone loss compared to vehicle (diluted DMOS) treatment [10]. Furthermore, in an in vitro osteogenesis study, we demonstrated that JTE013 treatment enhanced osteogenesis by promoting vesicle trafficking, Wnt /Ca^2+^, and bone morphogenetic protein (BMP)/Smad signaling in murine BMSCs cultured in osteogenic media [11]. However, it is unknown if inhibition of *S1PR2* by JTE013 can promote bone regeneration in animals following inflammatory bone loss.

Bones are highly vascularized organs with blood vessels that provide oxygen, nutrients, minerals, and secreted factors required for bone formation [12]. Many growth factors are involved in resolving inflammation, repairing tissues, and regenerating bone. These growth factors include vascular endothelial growth factor (*VEGF*) [12], platelet-derived growth factor (*PDGF*) [13], and growth differentiation factor 15 (*GDF15*) [14]. Because osteogenesis is coupled with angiogenesis, *VEGF* promotes bone repair [12] and delivery of exogenous *VEGF* promotes angiogenesis and the healing of bone defects [15,16]. In contrast, inhibition of *VEGF* activity by neutralizing *VEGF* receptor reduced angiogenesis, bone formation, and callus mineralization in femoral fractures [15]. *PDGF* is another growth factor expressed in tissues during bone fracture healing [17,18]. *PDGF* promotes bone healing because the growth factor is both chemotactic and mitogenic for osteoblast progenitor cells [19,20]; moreover, *PDGF* enhances angiogenesis [21]. Lastly, *GDF15*, a member of the transforming growth factor β (TGF-β) superfamily, promotes blood vessel growth by stimulating cell cycle progression, thus increasing bone healing [22]. In addition, *GDF15* has an anti-inflammatory role [23,24]. In this study, using a ligature-induced, periodontitis model, we aimed to determine if inhibiting *S1PR2* by JTE013 could increase gene expression for the growth factors (*VEGF*, *GDF15*, and *PDGF*), enhance angiogenesis, and promote alveolar bone regeneration following inflammatory bone loss.

## 2. Results

### 2.1. Treatment with JTE013 Enhanced VEGFA, PDGFA, and GDF15 in Murine BMSCs with or without Aggregatibacter Actinomycetemcomitans Infection

**Figure 2:**
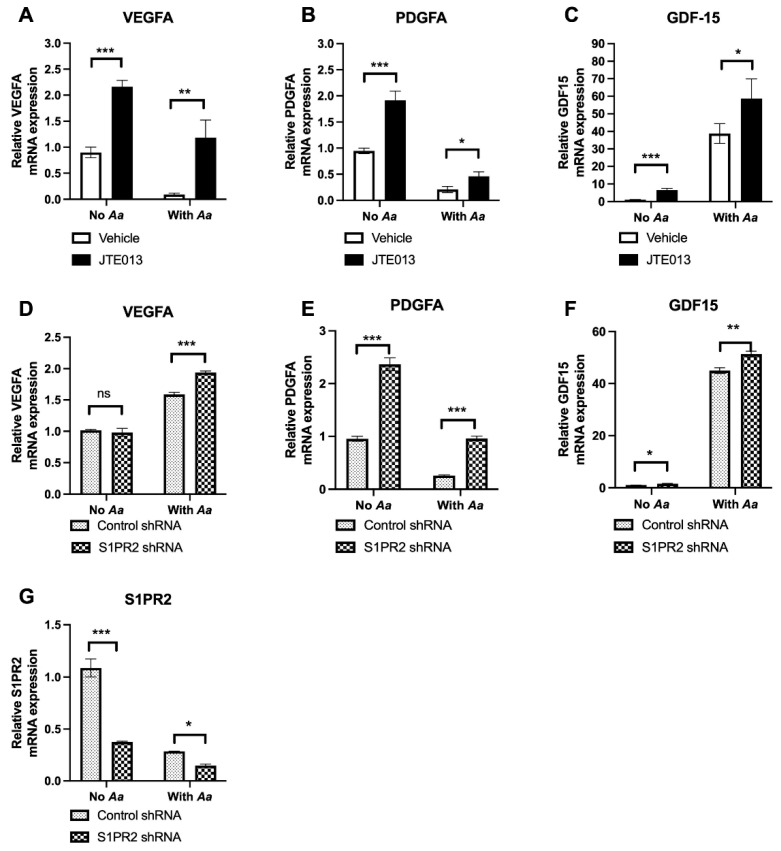
Effects of JTE013 or a *S1PR2* shRNA on *VEGFA*, *PDGFA*, and *GDF15* gene expressions in murine BMSCs infected with or without an oral bacterial pathogen *Aggregatibacter actinomycetemcomitans* (*Aa*). Murine BMSCs were cultured in DMEM media treated with vehicle (DMSO) or JTE013 (10 μM) for 30 min. Then, the cells were either uninfected or infected with Aa for 8 h. Another group of murine BMSCs were treated with a *S1PR2* shRNA or a control shRNA for 3 days with or without *Aa* infection for 8 h. The mRNA levels of (**A**,**D**) *VEGFA*, (**B**,**E**) *PDGFA*, (**C**,**F**) *GDF15*, and (**G**) *S1PR2* were evaluated by RT-q-PCR and normalized by *GAPDH* expression (*n* = 3, ns: no significance, * *p* < 0.05, ** *p* < 0.01, *** *p* < 0.001).

To determine the effects of inhibition of *S1PR2* by its specific inhibitor JTE013 on cell growth, we evaluated the mRNA levels of vascular endothelial growth factor A (*VEGFA*), platelet-derived growth factor subunit A (*PDGFA*), and *GDF15* in murine bone marrow stromal cells (BMSCs) that were treated with vehicle (diluted DMSO) or JTE013 with or without 8 h *Aggregatibacter actinomycetemcomitans* (*Aa*) infection. When murine BMSCs were treated with JTE013 for 8 h without *Aa* infection, the mRNA levels of *VEGFA*, *PDGFA,* and *GDF15* significantly increased by 2.4-fold, 2.0-fold, and 6.6-fold, respectively, when compared to vehicle controls (Figure 2A–C). In BMSCs treated with JTE013 and infected with *Aa* for 8 h, the mRNA levels of *VEGFA*, *PDGFA*, and *GDF15* significantly increased by 13.0-fold, 2.2-fold, and 1.5-fold, respectively, when compared to controls. These findings support that treatment with JTE013 enhanced *VEGFA*, *PDGFA*, and *GDF15* gene expression in BMSCs with or without *Aa* infection (Figure 2A–C).

To determine if treatment with JTE013 could potentially generate any off-target effects, murine BMSCs were treated with a *S1PR2* shRNA or a control shRNA and the cells were either uninfected or infected with *Aa* for 8 h. In BMSCs without *Aa* infection, no significant differences of *VEGFA* mRNA levels were found between cells treated with the *S1PR2* shRNA or the control shRNA (Figure 2D). However, treatment with the *S1PR2* shRNA significantly increased *PDGFA* and *GDF15* mRNA levels by 2.5-fold and 1.5-fold, respectively, compared to the control shRNA treatment (Figure 2E,F). In BMSCs infected with *Aa* for 8 h, treatment with the *S1PR2* shRNA significantly increased *VEGFA*, *PDGFA*, and *GDF15* mRNA levels by 1.2-fold, 3.7-fold, and 1.1-fold, respectively, when compared to controls (Figure 2D–F). The *S1PR2* shRNA reduced the mRNA level of *S1PR2* by 65.5% in murine BMSC without *Aa* infection and by 48.0% in murine BMSCs with *Aa* infection when compared to the control shRNA-treated BMSCs (Figure 2G). These results support that treatment with the *S1PR2* shRNA caused effects similar to treatment with JTE013 by promoting gene expression of *PDGFA* and *GDF15*. However, the JTE013 treatment also exhibited off-target effects, impacting *VEGFA* expression in BMSCs without *Aa* infection when compared to the *S1PR2* shRNA treatment.

### 2.2. Treatment with JTE013 Enhanced TGFβ/Smad and Akt Signaling in Murine BMSCs without Aggregatibacter Actinomycetemcomitans Infection

**Figure 3:**
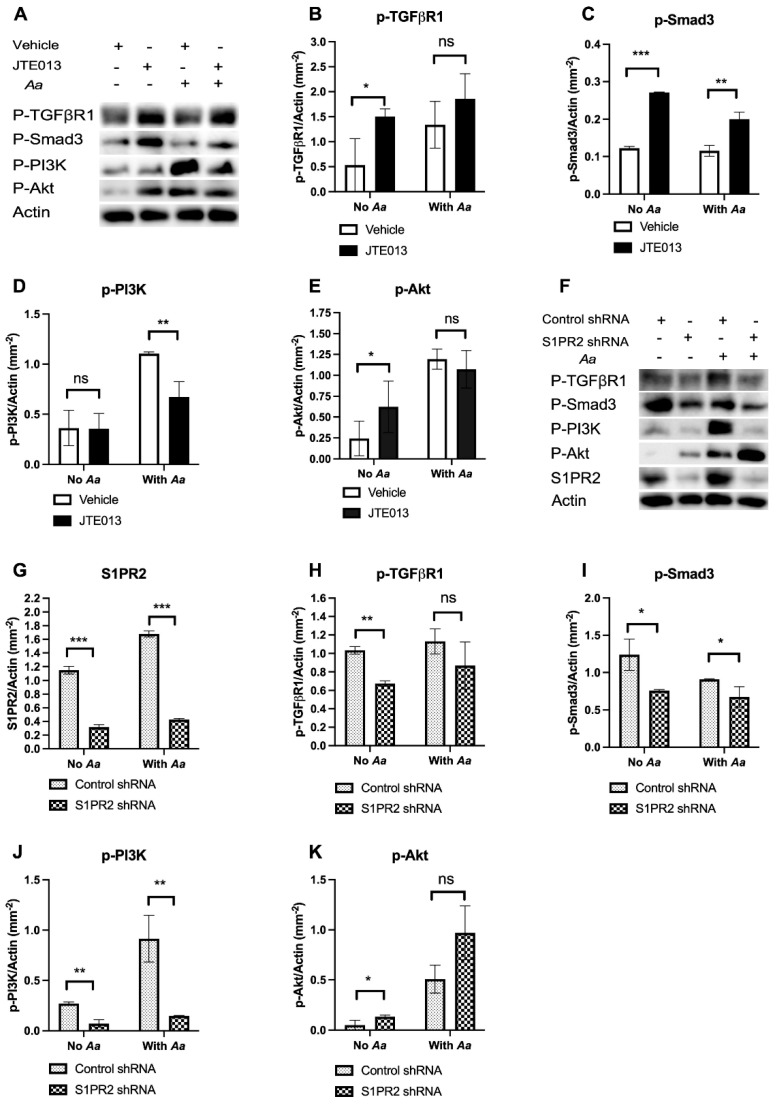
Effects of JTE013 or a *S1PR2* shRNA on TGFβ/Smad and PI3K/Akt signaling in murine BMSCs infected with or without an oral bacterial pathogen *Aggregatibacter actinomycetemcomitans* (*Aa*). Murine BMSCs were cultured in DMEM media pre-treated with vehicle (DMSO) or JTE013 (10 μM) for 30 min, or treated with a *S1PR2* shRNA or a control shRNA for 3 days. Then, the cells were either uninfected or infected with *Aa* for 8 h. (**A**,**F**) p-TGFβR1, p-Smad3, p-PI3K, p-Akt, and control actin protein levels in murine BMSCs. Protein densitometry of (**B**,**H**) p-TGFβR1, (**C**,**I**) p-Smad3, (**D**,**J**) p-PI3K, (**E**,**K**) p-Akt, and (**G**) *S1PR2* in murine BMSCs (*n* = 3, ns: no significance, * *p* < 0.05, ** *p* < 0.01, *** *p* < 0.001).

Many signaling pathways are involved in cell growth and bone regeneration, including TGFβ/Smad and PI3K/Akt signaling [25,26,27,28]. To determine which signaling pathways influence the release of growth factors in murine BMSCs, we evaluated p-TGFβ receptor 1 (p-TGFβR1), p-Smad3, p-PI3K, p-Akt, and control actin protein levels in murine BMSCs treated with vehicle or JTE013 with or without 8 h *Aa* infection. In uninfected murine BMSCs, treatment with JTE013 significantly increased p-TGFβR1 and p-Smad3 protein levels (Figure 3A–C). In murine BMSCs with *Aa* infection, treatment with JTE013 also increased p-TGFβR1 and significantly increased p-Smad3 levels compared to vehicle controls (Figure 3A–C). These findings support that JTE013 treatment promotes TGFβ/Smad signaling. In uninfected murine BMSCs, similar levels of p-PI3K were observed in murine BMSCs, regardless of treatment with vehicle or JTE013 (Figure 3A,D). Additionally, p-PI3K levels significantly decreased when JTE013-treated BMSCs were infected with *Aa* compared to vehicle controls (Figure 3A,D). In contrast, p-Akt protein levels significantly increased in uninfected murine BMSCs treated with JTE013 compared to controls (Figure 3A,E). Lastly, no significant differences of p-Akt levels were found in *Aa*-infected murine BMSCs treated with JTE013 or with vehicle (Figure 3A,E). These findings suggest that Akt signaling might be controlled by multiple signaling pathways. The interplay of PI3K and other signaling pathways perhaps affects p-Akt activity.

To determine if JTE013 treatment could generate off-target effects on TGFβ/Smad and PI3K/Akt signaling, we evaluated p-TGFβR1, p-Smad3, p-PI3K, p-Akt, and control actin protein levels in murine BMSCs treated with a *S1PR2* shRNA or a control shRNA. Treatment with the *S1PR2* shRNA significantly suppressed *S1PR2* protein levels compared to control shRNA treatment (Figure 3G). In uninfected murine BMSCs, treatment with the *S1PR2* shRNA significantly suppressed p-TGFβR1 and p-Smad3 protein levels compared to control shRNA treatment (Figure 3F,H,I). In shRNA-treated murine BMSCs that were infected with *Aa*, there was a reduction in p-TGFβR1 caused by the *S1PR2* shRNA; the *S1PR2* shRNA significantly suppressed p-Smad3 levels compared to control shRNA treatments (Figure 3F,H,I). These data suggest that JTE013 may have some off-target effects on TGFβ/Smad signaling compared to the *S1PR2* shRNA treatment. Although p-PI3K was significantly suppressed by treatment with *S1PR2* shRNA in murine BMSCs regardless of *Aa* infection (Figure 3J), the *S1PR2* shRNA significantly enhanced p-Akt in uninfected cells and increased p-Akt in *Aa* infected cells (Figure 3K). These results support that multiple signaling pathways might regulate Akt signaling in addition to PI3K.

### 2.3. Treatment with JTE013 Enhanced Alveolar Bone Regeneration following Inflammatory Bone Loss Induced by Ligature Placement in Mice

**Figure 4:**
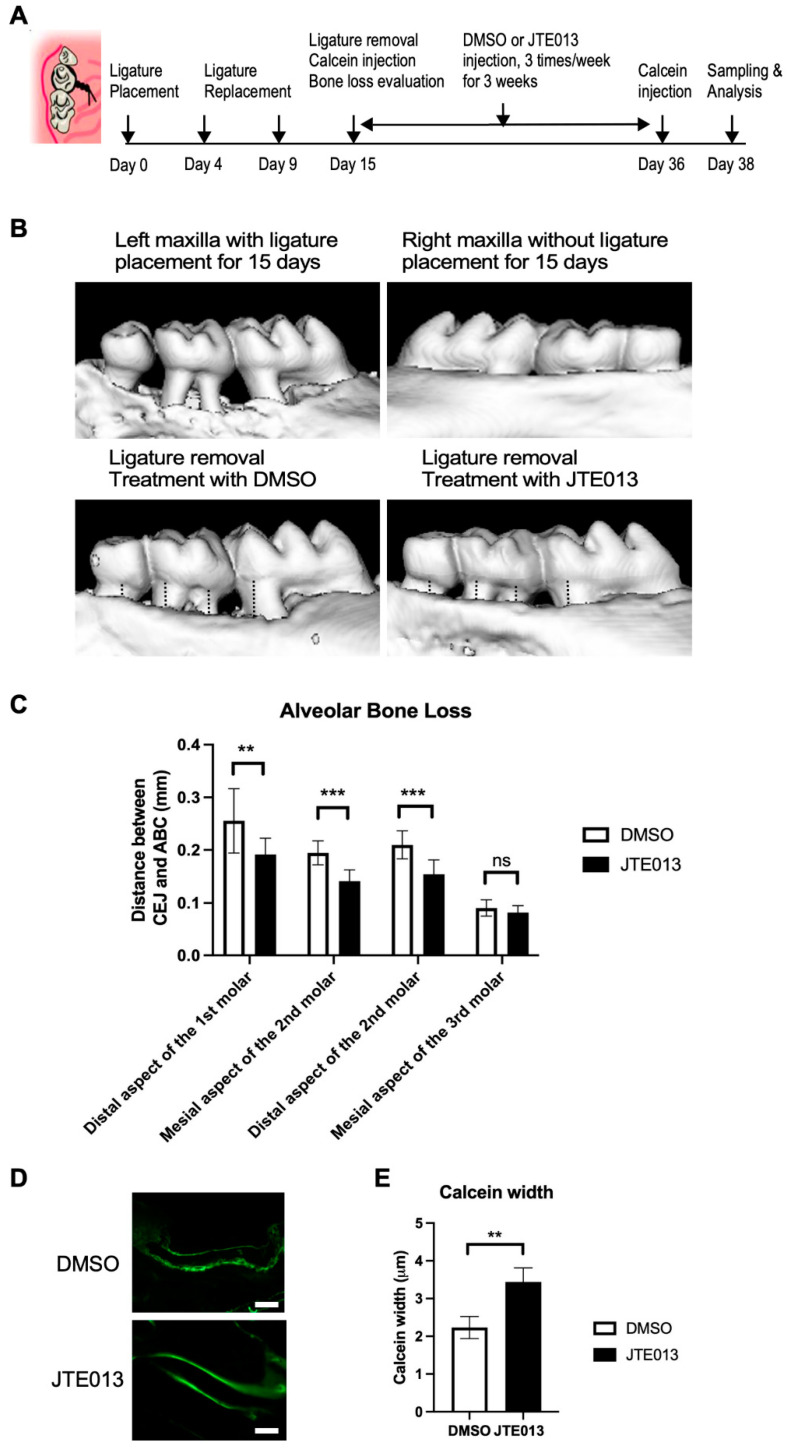
Treatment with JTE013 promoted alveolar bone regeneration following inflammatory bone loss. Eight-week-old male C57BL/6J mice were placed with ligatures for 15 days and removed on day 15, and calcein was injected to evaluate bone regeneration from day 15 to day 36 (3-week interval). Mice were treated with either diluted DMSO or JTE013 (20 μM) 3 times/week for 3 weeks (from day 15 to day 36). (**A**) Schematic diagram of mouse periodontitis model and treatment methods. (**B**) Representative images of micro-CT scanning of maxillary alveolar bone tissues are displayed. The dotted lines indicate the distances from cementoenamel junction (CEJ) to alveolar bone crest (ABC). (**C**) The distances between CEJ and ABC were quantified at the distal aspect of the 1st molar, the mesial aspect of the 2nd molar, the distal aspect of the 2nd molar, and the mesial aspect of the 3rd molar in the mice treated with DMSO or JTE013 (*n* = 10, ns: no significance, ** *p* < 0.01, *** *p* < 0.001). (**D**) Representative images of calcein in the periodontal tissues of mice treated with DMSO or JTE013. Pictures were taken under 200× magnification. The scale bars represent 50 μm. (**E**) The distances between two calcein lines (calcein width) were quantified in the DMSO-treated mice and in the JTE013-treated mice (*n* = 5, ** *p* < 0.01).

To determine if treatment with JTE013 could promote alveolar bone regeneration following inflammatory bone loss, we placed ligatures around the left maxillary 2nd molar for 15 days in C57BL/6J mice to induce alveolar bone loss. As expected, ligature placement caused severe alveolar bone loss in the distal aspect of the 1st molar, the mesial aspect of the 2nd molar, the distal aspect of the 2nd molar, and the mesial aspect of the 3rd molar areas compared to the right side maxilla without ligature placement (Figure 4B). After the ligatures were removed, mice were treated with JTE013 or vehicle (diluted DMSO) in the periodontal tissues 3 times/week for 3 weeks. Treatment with JTE013 significantly reduced alveolar bone loss, as measured by the distance from cementoenamel junction (CEJ) to alveolar bone crest (ABC), in the distal aspect of the 1st molar, the mesial aspect of the 2nd molar, and the distal aspect of the 2nd molar areas compared to DMSO treatment (Figure 4B,C). We also injected calcein (a fluorochrome that binds to calcium and can be incorporated at sites of mineralization [29]) twice (on day 15 and day 36) to measure bone regeneration after treating with JTE013 or vehicle. Calcein imaging in the periodontal tissues (Figure 4D) shows double calcein signaling, which reflected the two calcein injections during the 3-week interval. Treatment with JTE013 significantly increased calcein width (the distance between the two calcein signals in the periodontal tissues) compared to control (Figure 4D,E). These results support that treatment with JTE013 promoted alveolar bone regeneration following inflammatory bone loss.

### 2.4. Treatment with JTE013 Increased VEGFA, PDGFA, Osteocalcin, and Osterix mRNA Levels in Murine Gingival Tissues and Enhanced Angiogenesis in the Periodontal Tissues

**Figure 5:**
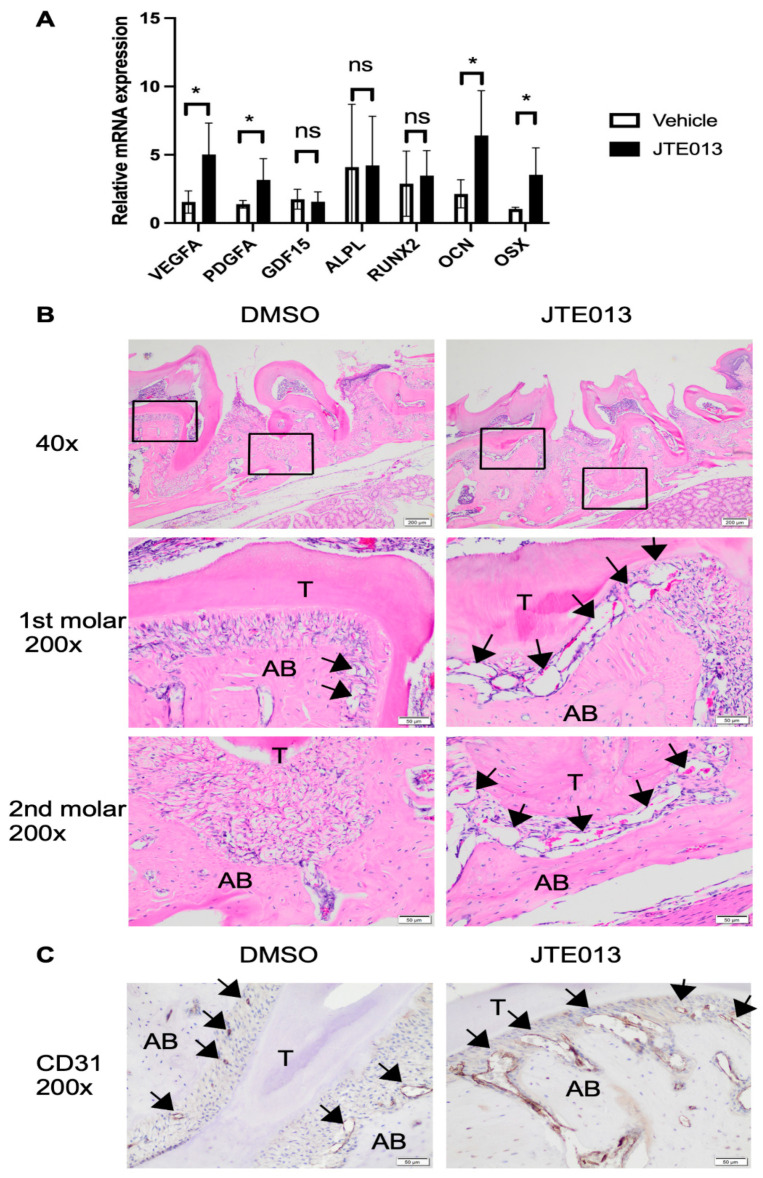
Treatment with JTE013 increased *VEGFA*, *PDGFA*, *OCN*, and *OSX* mRNA levels in the periodontal mucosa and enhanced angiogenesis in the periodontal tissues. (**A**) *VEGFA*, *PDGFA*, *GDF15*, *ALPL*, *RUNX2*, *OCN*, and *OSX* mRNA levels were evaluated by RT-q-PCR and normalized by *GAPDH* expression (*n* = 5, ns: no significance, * *p* < 0.05). (**B**) Representative images of hematoxylin and eosin (H&E) staining of the maxillary periodontal tissues of the DMSO-treated mice and the JTE013-treated mice. Images were taken under either 40× magnification or 200× magnification. (The black boxes indicate the magnified region near the 1st molar or 2nd molar. T stands for tooth. AB stands for alveolar bone. Black arrows indicate the dilated capillary-like structures around the alveolar bone tissues). (**C**) Representative images of CD31 staining of maxillary periodontal tissues of DMSO-treated mice and JTE013-treated mice. Images were taken under 200× magnification. (T stands for tooth. AB stands for alveolar bone. Black arrows indicate CD31-staining positive capillaries around the alveolar bone tissues).

In JTE013-treated periodontal tissues, we observed a 2.8-fold increase in *VEGFA* and a 2.4-fold increase in *PDGFA* mRNA levels compared to vehicle-treated controls (Figure 5A). However, no significant differences in *GDF15* mRNA levels were found between JTE013-treated animals and DMSO-treated animals. Because our previous study showed that JTE013 increased osteogenic genes, including alkaline phosphatase (*ALPL*), RUNX Family Transcription Factor 2 (*RUNX2*), osteocalcin (*OCN*), and osterix (*OSX*) in murine BMSCs cultured in osteogenic media [11], we also quantified these osteogenic gene mRNA levels in the periodontal tissues. In JTE013-treated oral mucosa, *OCN* and *OSX* mRNA levels were significantly enhanced compared to controls (Figure 5A). However, no significant differences in *ALPL* and *RUNX2* mRNA levels were observed between JTE013-treated group and DMSO-treated group. Hematoxylin & eosin (H&E) staining of periodontal tissues revealed that no inflammation was present in the periodontal tissues (Figure 5B). However, more dilated capillary-like structures were found around alveolar bone tissues in animals treated with JTE013 compared to animals treated with DMSO (Figure 5B). Additionally, immunohistochemical staining of CD31 (a marker of endothelial cells) in the periodontal tissues confirmed that treatment with JTE013 increased the presence of dilated CD31-staining positive capillaries around alveolar bone tissues when compared to animals treated with DMSO (which showed only small capillaries around alveolar bone tissues) (Figure 5C). Overall, our findings support that treatment with JTE013 enhanced *VEGFA*, *PDGFA*, *OCN*, and *OSX* gene expression and promoted angiogenesis in the periodontal tissues.

## 3. Discussion

In this study, we are the first to demonstrate that inhibition of *S1PR2* by JTE013 increased TGFβ/Smad and Akt signaling and significantly enhanced the mRNA levels of *VEGFA*, *PDGFA*, and *GDF15* in murine BMSCs. Using the ligature-induced periodontitis animal model, we are also the first to demonstrate that treatment with JTE013 enhanced *VEGFA*, *PDGFA*, *OCN*, and *OSX* mRNA levels; increased angiogenesis in the periodontal tissues; and promoted alveolar bone regeneration when compared to treatment with the control vehicle.

Previously, conflicting results were reported about how *S1PR2* regulates *VEGF* expression and angiogenesis. Inoki et al. [30] showed that both S1P and endothelial growth factor (*EGF*) stimulated the cell migration and the formation of capillary tube-like structures on the Matrigel using mouse vascular endothelial cells. The addition of the *S1PR2* specific antagonist (JTE013) further stimulated S1P and *EGF*-induced migration and capillary tube-like structure formation on the Matrigel [30]. Mechanistically, Inoki et al. [30] demonstrated that S1P and *EGF* enhanced Rac-GTP levels, and the addition of JTE013 further increased the Rac-GTP levels [30]. By implanting Matrigel in the subcutaneous tissues of mice, the researchers also demonstrated that treatment with JTE013 and S1P increased the number of infiltrating cells and the formation of blood vessels in mice [30]. In another preeclampsia (a pregnancy-induced hypertensive disorder) study, Zhang et al. [31] showed that treatment with JTE013 reduced blood pressure, attenuated inflammatory cytokines (TNF-α, IL-1β, and IL-6) in placental tissues, and significantly enhanced *VEGF* levels. However, Chumanevich et al. [32] reported that *S1PR2* knockout mice or treatment with JTE013 in cells (mouse bone marrow-derived mast cells and human skin mast cells) attenuated S1P-induced *VEGFA* levels. In another neuroblastoma study, Li et al. [33] showed that treatment with JTE013 inhibited tumor growth and *VEGF* mRNA expression. These differences might arise from the different type of cells being treated with JTE013. Different cells may express different levels of S1P receptors. We previously showed that JTE013 affected the protein expressions of multiple S1P receptors (S1PR1-S1PR5) [11]. Additionally, S1P receptors couple with multiple G proteins and influence multiple signaling pathways. The angiogenesis reaction might depend on the cell type, location of the receptors, and specific signaling pathways that are involved. Previously, we have showed that *S1PR2* is highly expressed in murine BMSCs, and treatment with JTE013 enhanced Rac1-GTP level compared to vehicle control [11]. In murine BMMs, treatment with JTE013 also significantly increased *VEGFA*, *PDGFA*, and *GDF15* compared with vehicle treatment regardless of *Aa* infection/ Our results are congruent with Inoki et al. [30] and Zhang et al. [31]’s studies [31] and support that JTE013 treatment promoted *VEGF* expression and angiogenesis. In the present study, we observed an increase in p-Akt in uninfected murine BMSCs treated with JTE013. Future studies are required to determine if Rac-GTP or other signaling pathways may also be involved in the activation of Akt.

In this study, we showed that treatment with JTE013 had some off-target effects via promoting TGFβ/Smad signaling and enhancing *VEGFA* expression in murine BMSCs even without *Aa* infection (Figure 2 and Figure 3). In contrast, treatment with the *S1PR2* shRNA in murine BMSCs suppressed TGFβ/Smad signaling and had no significant difference on *VEGFA* expression in uninfected BMSCs when compared to cells treated with the control shRNA (Figure 2 and Figure 3). The enhanced *VEGFA* in JTE013-treated cells might be associated with the increased TGFβ/Smad signaling. These results are consistent with our previous study [11], which showed that treatment with JTE013 increased BMP/Smad signaling (a member of TGFβ/Smad signaling), and treatment with the *S1PR2* shRNA in murine BMSCs inhibited BMP/Smad signaling.

Previous studies [34,35] demonstrated that activation of Smad signaling is affected by phosphorylation of MAPKs and glycogen synthase kinase-3 (GSK-3). This finding is significant because phosphorylation of MAPKs and GSK-3 can cause ubiquitination and proteasome-dependent degradation of regulatory Smad (R-Smad) [34,35]. We observed a reduction in p-Smad3 in murine BMSCs treated with JTE013 or the *S1PR2* shRNA and infected with *Aa* (Figure 3A,F) compared to JTE013 or the *S1PR2* shRNA-treated BMSCs without *Aa* infection. Perhaps this reduction in p-Smad is associated with enhanced phosphorylation of MAPKs induced by *Aa*, resulting in ubiquitination and degradation of R-Smad. We previously showed that treatment with JTE013 or *S1PR2* shRNA suppressed the phosphorylation of MAPKs induced by *Aa* [8]. Although treatment with *S1PR2* shRNA did not increase *VEGFA* in uninfected murine BMSCs, *S1PR2* shRNA did enhance *VEGFA* in BMSCs with *Aa* infection (Figure 2D). This result may have been caused by the down-regulation of phosphorylation of MAPKs by the *S1PR2* shRNA, perhaps reducing the degradation of R-Smad, thus subsequently increasing *VEGFA* expression.

**Figure 6:**
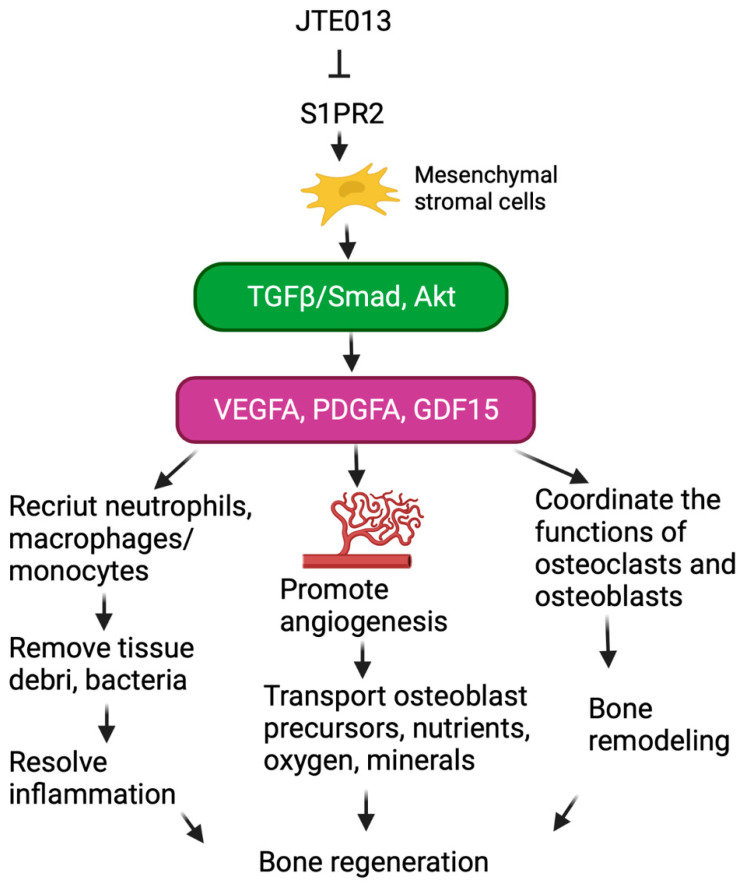
Schematic representation of the role of JTE013 in bone regeneration. Treatment with a *S1PR2* antagonist (JTE013) in mesenchymal stromal cells increases TGFβ/Smad and Akt signaling and enhances *VEGFA*, *PDGFA*, and *GDF15* gene expressions, subsequently promoting inflammation resolution, angiogenesis and bone mineralization, and bone remodeling processes.

Bone regeneration undergoes three continuing and overlapping phases, including inflammation, regeneration, and remodeling [36]. The resolution of inflammation involves recruiting neutrophils and macrophages/monocytes to the injury sites to remove tissue debris and microbial pathogens. *VEGF* plays an important role in the inflammation phase during bone repair by attracting neutrophils and macrophages [37,38,39], facilitating the resolution of inflammation (Figure 6). During bone regeneration phase, both *VEGF* and *PDGF* regulate the migration and proliferation of endothelial cells, and control blood vessel permeability [19,37]. Additionally, *VEGF* and *PDGF* stimulate the migration and proliferation of osteoblast progenitors, and promote the differentiation of osteoblasts [19,37]. Furthermore, the increased angiogenesis subsequently provides osteoblast progenitors, nutrients, oxygen, and minerals required for bone mineralization [37]. A previous study [15] demonstrated that *VEGF* is critical for bone repair because mice treated with a neutralizing *VEGF* receptor antibody reduced angiogenesis, bone formation, and callus mineralization [15]. In contrast, treatment with an exogenous *VEGF* enhanced blood vessel formation, ossification, and new bone maturation in animals [15]. In the bone remodeling phase, *VEGF* and *PDGF* influence the function of both osteoclasts and osteoblasts, coordinating them to replace woven bone (characterized by a haphazard organization of collagen and mechanically weak) with lamellar bone (characterized by parallel alignment of collagen into sheets and mechanically strong) [21,37]. Thus, *VEGF* and *PDGF* play essential roles in the inflammation resolution phase, bone regeneration phase, and bone remodeling phase (Figure 6). *GDF15* is another cytokine that promotes angiogenesis as demonstrated by Wang et al. [22]. *GDF15* enhanced the expression of cyclins D1 and E and promoted the proliferation of human umbilical vein endothelial cells (HUVECs) [22]. Akt was also identified as one of the signaling pathways that induces the production of *GDF15* [22]. Treatment with *GDF15* promoted neovascularization in the critical-sized calvarial defects of mice compared to sterile saline treatment [22]. In this study, although JTE013 significantly enhanced *GDF15* in vitro compared to vehicle treatment in murine BMSCs (Figure 2C), no significant differences of *GDF15* mRNA levels were found in the oral mucosa between JTE013-treatment animals and DMSO-treated animals (Figure 5A). Perhaps this finding can be attributed to the different harvest time of cells between the in vitro study (8 h after treatment) and the in vivo study (2 days after JTE013 treatment). Future studies are required to determine if treatment with JTE013 could increase *GDF15* mRNA levels within 8 h of treatment.

We previously showed that murine BMSCs treated with JTE013 increased vehicle trafficking when cultured in osteogenic media [11]. Treatment with JTE013 also enhanced the mRNA levels of osteogenic genes (*ALPL*, *RUNX2*, *OCN*, and *OSX*) in murine BMSCs cultured in osteogenic media [11]. The increases of these osteogenic genes were associated with the enhanced intracellular vesicle trafficking in BMSCs cultured in osteogenic media [11]. In murine BMSCs cultured in DMEM media, JTE013 did not enhance vehicle trafficking when compared to DMSO-treated controls. Because the osteogenic media are a DMEM media supplemented with β-glycerophosphate, dexamethasone, and ascorbic acid, the enhanced vesicle trafficking in JTE013-treated cells might be associated with *S1PR2* in response to stimulation with β-glycerophosphate, dexamethasone, or ascorbic acid. In the current study, we only observed a significant increase in *OCN* and *OSX* mRNA levels in the periodontal tissues (Figure 5A), which was potentially associated with increased angiogenesis in periodontal tissues, and subsequently promoting bone matrix regeneration and calcification. In the periodontal tissues, we also observed significant increases in *VEGFA* and *PDGFA* mRNA levels in mice treated with JTE013. Because *VEGF* and *PDGF* promote the resolution of inflammation, angiogenesis, bone regeneration, and the bone remodeling process (Figure 6), treatment with JTE013 increased alveolar bone regeneration following ligature removal.

The following limitations occurred in the present study. First, we only harvested periodontal oral mucosa to analyze the mRNA levels of *VEGFA*, *PDGFA*, *GDF15*, *ALPL*, *RUNX2*, *OCN*, and *OSX*. Because oral mucosa is the top layer of periodontal tissues, it may not reflect overall gene expression in the entire periodontal tissues. Future studies are needed to determine if treatment with JTE013 could increase these gene expressions in the entire periodontal tissues. Second, because *S1PR2* couples with multiple G proteins, we could not determine which G proteins are associated with the activation of TGFβ/Smad and Akt signaling. Future studies are required to determine which G proteins are involved in the activation of TGFβ/Smad and Akt signaling. Finally, images of H&E staining of periodontal tissue sections revealed similar alveolar bone morphology between JTE013-treated mice and vehicle-treated mice. Future studies using an electronic microscope are needed to display more detailed structures of regenerated bone tissues.

In summary, the present study is the first to demonstrate that treatment with the *S1PR2* antagonist (JTE013) enhanced TGFβ/Smad and Akt signaling and increased *VEGFA*, *PDGFA*, and *GDF15* gene expressions in murine BMSCs with or without bacterial infection. Using a ligature-induced periodontitis animal model, we are the first to demonstrate that treatment with JTE013 promoted angiogenesis and alveolar bone regeneration following inflammatory bone loss. Because treatment with JTE013 inhibited inflammation, attenuated osteoclastogenesis, promoted angiogenesis, and induced bone healing, inhibition of *S1PR2* by JTE013 may potentially serve as a therapeutic strategy to treat inflammatory bone loss diseases, including periodontitis.

## 4. Materials and Methods

### 4.1. Animals, Cells, and Reagents

Eight-week-old male C57BL/6J mice were purchased from Jackson Laboratory (Bar Harbor, ME, USA). Mice were housed under a 12 h light/12 h dark cycle in specific pathogen-free conditions and had free access to food and water. All animal-related work was conducted in accordance with the guidelines laid down by the National Institute of Health (NIH) in the United States regarding the usage of animals for experimental procedures and approved by the Institutional Animal Care and Use Committee at the Medical University of South Carolina. The murine BMSCs were harvested from 8-week-old C57BL/6J mice by flushing bone marrow cells from femur and tibia. To separate BMMs from BMSCs, bone marrow cells were plated in 15 cm cell culture dishes and incubated at 37 °C with 5% CO_2_ for 3 days. The suspended BMMs were removed and discarded. After washing plate with PBS, the attached murine BMSCs were cultured in Dulbecco’s Modified Eagle Medium (DMEM) and supplemented with 10% fetal bovine serum (FBS), 100 U/mL penicillin, and streptomycin. JTE013 was purchased from Cayman Chemical (Ann Arbor, MI, USA) and dissolved in DMSO as 20 mM stock solution. DMEM, FBS, penicillin, and streptomycin were purchased from Fisher Scientific (Suwanee, GA, USA). Calcein was obtained from Sigma Aldrich (St. Louis, MO, USA) and dissolved in 2% bicarbonate as 4 mg/mL stock solution.

### 4.2. Generation of ShRNA Lentivirus

The *S1PR2* shRNA and control shRNA were generated as previously described [8]. Briefly, human embryonic kidney (HEK) 293 cells were co-transfected with *S1PR2* shRNA plasmid DNA or control shRNA plasmid DNA along with lentiviral packaging plasmids pCMV-VSV-G and pCMV-dR8.2 dvpr (Addgene, Cambridge, MA, USA) using lipofectamine 2000 (Life Technologies). Furthermore, 3 days after transfection, the supernatant was collected and ultracentrifuged at 25,000 rpm for 1.5 h at 4 °C using a Beckman Ultracentrifuge (Beckman Coulter, Indianapolis, IN, USA). The viral pellet was resuspended in serum-free DMEM medium, and viral titer was determined with a HIV-1 p24 Antigen ELISA kit (Zeptometrix, Buffalo, NY, USA).

### 4.3. Infection with Aggregatibacter Actinomycetemcomitans

*Aggregatibacter actinomycetemcomitans* (*Aa*, ATCC 43718) was purchased from American Type Culture Collection (Manassas, VA, USA), grown on Difco^TM^ brain heart infusion agar plates (BD Biosciences, Sparks, MD, USA), and cultured in Bacto^TM^ brain heart infusion broth (BD Biosciences) for 24 h at 37 °C with 10% CO_2_. The bacteria were centrifuged, washed with PBS with 5% glycerol, and resuspended in PBS with 5% glycerol. We determined the bacterial concentration by measuring bacterial optical density and by bacterial plating on brain heart infusion agar plates (OD_600_ = 1, about 3 × 10^7^ colony forming unit, CFU/mL). Murine BMSCs were pre-treated with DMSO or JTE013 (10 μM) for 30 min, and then the BMSCs were either uninfected or infected with *Aa* (1 CFU/cell) for 8 h.

### 4.4. RNA Extraction, Reverse Transcription, and Quantitative Polymerase Chain Reaction (RT-qPCR)

Total RNA was isolated from cells using TRIzol (Life Technologies, Carlsbad, CA, USA), according to the manufacturer’s instructions. The complementary DNA was synthesized with a TaqMan reverse transcription kit (Life Technologies) using the total RNA (1 μg). Real-time PCR was performed using a StepOnePlus Real-Time PCR System (Life Technologies) as previously described [8]. The following amplicon primers were obtained from Life Technologies: *VEGFA* (Mm00437306_m1), *PDGFA* (Mm01205760_m1), *GDF15* (Mm00442228_m1), *ALPL* (Mm00475834_m1), *RUNX2* (Mm00501584_m1), *OCN* (also called bone gamma carboxyglutamate protein *BGLAP*, Mm03413826_mH), *OSX* (also called *Sp7* transcription factor, Mm04209856_m1), *S1PR2* (ARFVPA4), and *GAPDH* (Mm99999915_g1). Amplicon concentration was determined using threshold cycle values compared with standard curves for each primer. Sample mRNA levels were normalized to control *GAPDH* expression and expressed as fold changes as compared to control groups.

### 4.5. Protein Isolation and Western Blot Analysis

Proteins were extracted using RIPA cell lysis buffer (Cell signaling Technology, Danvers, MA, USA). The protein concentration was determined with a DC^TM^ protein assay kit (Bio-Rad Laboratories, Hercules, CA, USA). Proteins were loaded on 10% Tris-HCl gels and electro-transferred to nitrocellulose membranes. Membranes were blocked with milk for 1 h at RT and incubated with primary antibody overnight at 4 °C. The p-TGFβR1 (PA5-40298) antibody was obtained from Thermo Fisher Scientific (Waltham, MA, USA). The antibodies to p-Smad3, p-PI3K, p-Akt, p-38, and pan-actin were purchased from Cell Signaling Technology (Danvers, MA, USA). The *S1PR2* antibody (SAB4503614) was purchased from Sigma Aldrich (St. Louis, MO, USA). All primary antibodies were incubated at 1:500 or 1:1000 dilution overnight at 4 °C. After washing, the nitrocellulose membranes were incubated at room temperature (RT) for 1 h with horseradish peroxidase-conjugated secondary antibodies (Cell Signaling Technology) and developed using SuperSignal West Pico Chemiluminescent Substrate (Life Technologies Grand Island, NY, USA). Digital images were recorded with a G-BOX chemiluminescence imaging system (Syngene, Frederick, MD, USA). Protein densitometry was analyzed with the GeneTools software (Syngene).

### 4.6. Animal Treatment

To induce inflammatory bone loss, 8-week-old male C57BL/6J mice (*n* = 30) were placed with 5.0 silk sutures (Roboz Surgical Instrument Co., MD, USA) around the cervical region of left maxillary second molars under isoflurane anesthesia. The right maxillary teeth were untreated to serve as a baseline control. The ligatures were checked daily and remained in place in all mice during the experimental period. All the ligatures were replaced on day 4 and day 9 to induce minor tissue injury, bacterial colonization, and persistent inflammatory bone loss response. The animals were divided into 3 groups (10 mice/group). The first group of mice were sacrificed on day 15 after ligature placement and both sides of maxillary tissues were harvested to evaluate alveolar bone loss. The ligatures were removed on day 15 for the remaining mice and calcein (20 mg/kg) was injected intraperitoneally (i.p.). The remaining 2 groups of mice were injected with either 8 μL of diluted DMSO (*n* = 10) or 8 μL of JTE013 (20 μM, *n* = 10) in the lingual periodontal mucosal tissues 3 times/week for 3 weeks. Three weeks after JTE013 or DMSO treatment, the mice were injected with calcein again. Two days after calcein injection, the mice were euthanized. Left lingual oral mucosal tissues (5 mice/group) were harvested and stored in TRIzol reagent (ThermoFisher Scientific, Waltham, MA, USA). Tissues were then homogenized in TRIzol by a bullet blender tissue homogenizer (Next Advance, Inc. Troy, NY, USA) with RNase-free stainless steel beads. Both sides of maxillary tissues (10 mice/group) were fixed in 10% buffered formalin solution for 48 h and later stored in 70% ethanol.

### 4.7. Micro-Computed Tomography (Micro-CT) Scanning and Alveolar Bone Loss Assessment

Maxillary tissues were scanned with a cone-beam µ-CT40 system (Scanco Medical AG, Switzerland). Three dimensional micro-CT images were visualized with the GE Healthcare MicroView software using the isosurface function (with image threshold 7000 and surface quality factor 0.5). The alveolar bone loss was assessed by measuring the distance from cementoenamel junction (CEJ) to alveolar bone crest (ABC) using Adobe Photoshop CS5.1 software. The distance was calibrated by the height of second crown.

### 4.8. Tissue Processing and Staining

Half of the maxillary bone tissues (5/group) were decalcified in a 20% EDTA solution for 4 weeks followed by paraffin embedding. Five µm sagittal paraffin tissue sections were cut, stained with hematoxylin and eosin (H&E) for general histology, and evaluated by an experienced pathologist. The other half of the maxillary tissues (5/group) were processed for methyl methacrylate embedding and ground sectioning to evaluate calcein signaling in the tissues. For immunochemical staining of CD31, the 5 μm paraffin sections were deparaffinized in xylene and rehydrated in graded alcohol series. The sections were incubated in Tris-EDTA buffer for epitope retrieval. After washing with TBST solution 3 times, the slides were blocked with 10% goat serum for 1 h. Then, the slides were incubated with anti-CD31 rabbit monoclonal antibody (Abcam EPR17259, 1:2000) overnight at 4 °C. After washing, the slides were incubated with 3% H_2_O_2_ for 15 min at RT to inhibit endogenous peroxidase. After washing, the slides were incubated with goat anti-rabbit biotinylated 2nd antibody (Vector Laboratories, Newark CA, USA, 1:200) for 1 h at RT. The slides were washed and incubated with standard avidin–biotin complex (Vector Laboratories) for 30 min at RT. Antibody binding was revealed using H_2_O_2_ as a substrate and diaminobenzidine as chromogen (Vector Laboratories). Counterstaining was performed with hematoxylin. H&E and CD31 images were recorded with an Olympus BX43 microscope and calcein images were recorded with a Zeiss Axio Imager A1 Epifluorescence microscope.

### 4.9. Statistical Analysis

Data were analyzed by unpaired t-test with Welch’s correction. All statistical tests were performed using GraphPad Prism software (GraphPad Software Inc., La Jolla, CA, USA). Values are expressed as means ± standard error of the means (SEM) of 3 independent experiments. A *p* value of 0.05 or less was considered significant.

## Data Availability

The data presented in this study are available upon request from the corresponding author.
